# Expanding Contraceptive Method Choice With a Hormonal Intrauterine System: Results From Mixed Methods Studies in Kenya and Zambia

**DOI:** 10.9745/GHSP-D-20-00556

**Published:** 2021-03-31

**Authors:** Deborah Sitrin, Anne Pfitzer, Gathari Ndirangu, Ameck Kamanga, Brenda Onguti, Susan Ontiri, Jully Chilambwe, Victor Kabwe, Lola Aladesanmi, Leah Elliott, Neeta Bhatnagar

**Affiliations:** a Maternal and Child Survival Program, Jhpiego, Washington, DC, USA.; b Maternal and Child Survival Program, Jhpiego, Nairobi, Kenya.; c Safe Motherhood 360+, Jhpiego, Lusaka, Zambia.; d Maternal and Child Survival Program, Jhpiego, Lusaka, Zambia.

## Abstract

**Introduction:**

Few women in low- and middle-income countries have access to the hormonal intrauterine system (IUS). Past research from a small number of facilities and the private sector suggest the IUS could be an important addition to the contraceptive method mix because it is the only long-acting method some women will adopt and users report high satisfaction and continuation. We aimed to determine whether these promising results were applicable in public facilities in Kenya and Zambia.

**Methods:**

We used a mixed-methods approach with program monitoring data, interviews with women who received an IUS, and qualitative focus group discussions with providers. Data were collected in 2017–2019.

**Results:**

Facilities in Kenya and Zambia reported 1,985 and 428 IUS insertions, respectively. If the IUS had not been available, 30% of adopters would have chosen a short-acting method. Women and providers gave diverse reasons for adopting the IUS, with the desire for fewer side effects being frequently mentioned in focus group discussions. Many IUS adopters first heard of the method on the day it was inserted (70% in Kenya, 47% in Zambia), yet providers reported that many women were unwilling to try a method they were just hearing about for the first time. Satisfaction and continuation were high: 86% of adopters in Kenya were still using the method 3–6 months after insertion and 78% were in Zambia (average 10 months post insertion). Providers also reported that most IUS adopters were satisfied; they rarely returned with complaints that could not be addressed with additional counseling.

**Conclusion:**

Expanding IUS access through the public sector shows promise to increase contraception use and continuation in low- and middle-income countries. Efforts to strengthen availability should consider demand and engage directly with various communities, including youth, around availability of a new long-acting option.

## Abstract (precis)

Although the hormonal intrauterine system has limited availability in low- and middle-income countries, this highly effective long-acting reversible contraceptive method has the potential to be an important addition to the method mix. Introduction of the method in the public sector under “real-world” conditions in Kenya and Zambia shows promise to increase contraception use and continuation.

## Teaser

**Key Messages:**

- Making the hormonal intrauterine system (IUS) available in public facilities in low-income countries could increase uptake of long-acting contraception because it appeals to some women who would not otherwise choose a long-acting method; 30% of hormonal IUS adopters would have chosen a short-acting method if the hormonal IUS had not been available.
- Satisfaction and continuation rates were high among interviewed hormonal IUS adopters. Providers also reported that most hormonal IUS adopters were satisfied and rarely returned with complaints that could not be addressed with additional counseling.
- Providers reported that many women were not willing to try a method they were hearing about for the first time.

**Key Implications:**

- When taking steps to increase the availability of the hormonal IUS, donors and policy makers must fund demand-creation efforts to increase awareness of a new long-acting contraceptive option that has characteristics that are distinctly different from other long-acting and short-acting methods.
- Program managers should develop introduction and scale-up plans that allow for ongoing support and mentorship of providers in offering a new long-acting contraceptive method.

## BACKGROUND

The hormonal intrauterine system (IUS) is a highly effective, long-acting reversible contraceptive (LARC) method with numerous noncontraceptive benefits.^1^^–^^3^ The hormonal IUS has been very successful in high-income countries; in the United States, it is more widely used than all other contraceptive methods introduced in recent decades.^4^ However, the hormonal IUS has limited availability in low- and middle-income countries (LMICs), primarily due to the cost of branded products. Accessibility in LMICs could increase in the near future with the introduction of new low-cost hormonal IUS products, such as Avibela from Medicines360, plus increasing awareness of a generic product available for donation through the International Contraceptive Access (ICA) Foundation.^5^^,^^6^

The hormonal IUS has the potential to be an important addition to the method mix in LMICs. Generally, increasing the number of available methods increases contraceptive use, although adding a method that is a variation of existing methods may have a greater effect on continuation than uptake.^7^ The hormonal IUS is a small, flexible T-shaped plastic frame with a white cylinder-shaped, hormone-filled vertical stem with 2 nylon threads at the end, and it has specific attributes that make it attractive. Use of a hormonal IUS often reduces menstrual bleeding and cramping (nonhormonal copper-containing intrauterine devices [IUDs] can have the opposite effect), and it releases less hormone into the bloodstream than other hormonal methods.^8^ These features are important because the most common reasons that women in LMICs cite for discontinuing contraception or not using it at all are side effects and health concerns.^9^^,^^10^

Past research on the hormonal IUS in LMICs suggests women will choose it when it is offered as part of a mix of methods.^11^^–^^13^ For some women, it may be the only long-acting method they will adopt when given the option of IUDs and implants.^11^^,^^13^ Hormonal IUS users also report high satisfaction and continuation of the method.^13^^–^^15^ Although these results suggest the hormonal IUS could be an important addition to the contraceptive method mix, the evidence has limited generalizability to the public health care sector, which remains an important source of contraception in LMICs.^16^ Participants in past hormonal IUS studies in LMICs were mostly recruited from clinics run by nongovernmental organizations, social franchises, or outreach services or from a very small number of public facilities. Two studies involved the same cohort recruited from 1 public facility in Kenya.^11^^,^^14^ A recent study in Nigeria gathered data on the hormonal IUS provided via social franchise, mobile outreach, and 20 public sector providers, but the latter inserted too few hormonal IUSs to be included in the analysis.^13^ A study in Ghana involved 12 providers operating in 6 hospitals where IUD acceptance was already high.^15^ Providers that did counseling and insertion in these studies were usually experienced in IUD provision. Provider knowledge, experience, and comfort with contraceptive methods affect the quality of counseling, which in turn affects women's contraceptive choice and continuation.^17^^,^^18^ Evidence for the viability of the hormonal IUS in public sector health facilities, including lower level facilities, in LMICs is not yet sufficient.

The U.S. Agency for International Development (USAID) flagship Maternal and Child Survival Program (MCSP) aimed to fill this evidence gap by introducing the hormonal IUS into public facilities in 2 counties in Kenya and 4 provinces in Zambia. MCSP used donated products from the ICA Foundation with implementation activities executed in partnership with the USAID-funded Afya Halisi project^19^ in Kenya and Safe Motherhood 360+ project (SM360+)^20^ in Zambia. Introduction started in late 2016 in Kenya and early 2017 in Zambia.

This article explores characteristics of women adopting the hormonal IUS at public facilities with comparison to IUD adopters, reasons women chose the hormonal IUS, sources of information about the method, user satisfaction, and continuation rates. Data collected from women were triangulated with provider perspectives. MCSP's intent was to learn whether promising results from earlier studies on the hormonal IUS in LMICs hold true when the method is introduced in the public sector under “real-world” conditions. This evidence contributes to the learning agenda developed by a global Hormonal IUS Consultative Group of donors, implementers, and suppliers.^21^

This study investigated whether promising results from earlier studies hold true when the hormonal IUS is introduced in the public sector under “real-world” conditions.

## PROGRAM DESCRIPTION

In Kenya and Zambia, MCSP consulted with Ministry of Health (MOH) stakeholders to plan and design the program, including tools for on-the-job training with ongoing mentorship, an approach shown to be cost efficient for building provider skills.^22^^,^^23^ MCSP used its LARC Learning Resource Package, a modular set of training materials that focuses on hands-on practice for developing clinical LARC skills (Box).^24^ The training approach and materials align with the current or anticipated national MOH plans for expanding LARC access in both countries.

BOX **Capacity-Building Approach Used by the Maternal and Child Survival Program for Hormonal Intrauterine System Training**

Traditional training through extended, off-site, group-based workshops often fails to improve health care provider performance.^25^^,^^26^ Sustained improvements are better achieved through interactive techniques, simulated practice, immediate feedback, and ongoing learning opportunities delivered at appropriate doses and frequencies.^26^

In light of this evidence, and to ensure hormonal intrauterine system (IUS)/long-acting reversible contraceptive (LARC) training translated into performance, the Maternal and Child Survival Program (MCSP) used an on-the-job, modular (no session longer than 3 hours) approach to help build and strengthen the competency and confidence of providers to learn and perform essential job skills with minimal disruption to services. Training was interactive and held at health care facilities with special attention given throughout to learning and practicing skills through role plays and simulations (i.e., with anatomical models) with family planning clients. Training content was tailored at each site based on identified learning needs of providers, using the appropriate modules from MCSP's LARC Learning Resource Package as needed. For example, in Kenya, the copper intrauterine device (IUD) module was used in addition to the hormonal IUS and postpartum modules when LARC providers were not sufficiently proficient in IUD counseling and insertion, so providers could confidently and competently provide both the IUD and hormonal IUS. The LARC Learning Resource Package contains 5 clinical modules plus modules on family planning counseling and assessing medical eligibility (with activities designed to help providers strengthen their communication skills to enable clients to make well informed and voluntary decisions), infection prevention, and quality care.

Mentoring complements on-the-job-training by supporting skill transfer and service implementation after training, ensuring that remaining skill gaps of learners are addressed during post-training mentorship sessions. MCSP defined mentoring as “the process through which an experienced and empathetic person who is proficient in her/his content area (a mentor) teaches and coaches another individual (mentee) or group of individuals (mentees), in person and/or virtually, to ensure competent workplace performance and provide ongoing professional development.”^27^ As part of hormonal IUS introduction, mentors supported ongoing practice and quality improvement activities to reinforce learning and facilitate application of new skills during clinical practice. When needed (particularly if mentors offered training at a facility where they were not based), mentors sometimes identified high-performing mentees who served as peer practice coordinators within their facilities, supporting their peers in learning and practice between clinical mentorship sessions. Groups of mentors and mentees also established WhatsApp groups to facilitate communication at a distance and rapid response to questions. As a result, mentees received continuous feedback and reinforcement on their performance even when a mentor was not present. Once mentees were confident in providing hormonal IUS services, they could then be assessed and certified. In addition, in Zambia, district-level supervisors provided general technical supervision.

To implement cascade training, MCSP trained providers (mainly nurses and midwives) who had been identified as MOH mentors to support other providers in building their clinical skills. The first step of the process was to train the mentors in hormonal IUS counseling and insertion and removal techniques and to review the process for training and mentoring other providers. After mentors conducted 10 insertions in Kenya (as many as possible under observation during training and mentorship and the remainder done independently and logged) and 4 insertions in Zambia (2 while being coached and 2 under observation during training or mentorship), they underwent assessment to become certified hormonal IUS providers. After becoming certified, mentors were then expected to train and provide ongoing support for other providers (mentees) in facilities where they worked and/or nearby facilities, as well as continue to conduct insertions. Mentees were other providers (also mainly nurses and midwives) selected collaboratively by MOH coordinators, the project team, and facility in-charges. On-site training of mentees was spread over 4 consecutive days and lasted no more than 3 hours per day. Mentors then developed a follow-up mentoring plan based on the performance of the mentees. The criteria for becoming a certified hormonal IUS provider were the same for mentees as for mentors (Box).

In Kenya, the mentors had been previously trained by MCSP in other LARCs (implants and IUDs) in 2016 but were inexperienced in hormonal IUS. With hormonal IUS introduction, MCSP led additional training for mentors on hormonal IUS plus postpartum insertion (for the IUS and IUD) in December 2016, using the appropriate modules from the Learning Resource Package. Additional mentors were trained in 2017 on all LARCS under the Afya Halisi project. As of mid-2019, 65 mentors were trained in hormonal IUS with 48 certified plus 190 mentees trained by mentors, although none were certified due to delays in rolling out the assessments. (The assessments were costly because they involved travel for 3 assessors to each site, so the process was delayed waiting for more providers to reach the 10-insertion threshold for certification. In the meantime, providers continued to offer the service, even if they exceeded the threshold.)

In Zambia, the SM360+ project trained maternity care providers to become LARC providers and mentors from February to August 2017, with MCSP contributing materials and trainers to incorporate the hormonal IUS into the training. The mentors had previous training in maternal and newborn health and mentorship skills, but were often inexperienced with LARCs. They were expected to start integrating LARC counseling and provision into their maternity clinical work and mentorship of other providers of maternity services. (Although the project targeted maternity providers, facilities rotated providers and some reported working in different departments during the study period.) As of mid-2019, 68 mentors were trained in all LARCs including hormonal IUS (17 in Luapula, 20 in Eastern, 8 in Central, 23 in Southern Province) with 49 certified plus 134 mentees trained by mentors with 91 certified. In both countries, the number of trained and certified providers continues to rise as cascade training is ongoing.

Mentors and mentees worked in facilities that offered short-acting contraceptive methods as well as IUDs and implants (some also provided sterilizations), although temporary stock-outs or staffing disruptions sometimes limited availability of certain methods. Hormonal IUS was offered free of charge, as are all contraceptive methods in the public sector in these countries. MCSP distributed donated ICA Foundation commodities directly to facilities. Providers in both countries gave information on the hormonal IUS during group talks at facilities and one-on-one counseling. Information on the hormonal IUS was incorporated into discussions or counseling on family planning and the various methods available, and not done separately. Providers had been trained to give a woman more information on the characteristics of a method once she made her choice, including expected side effects, bleeding changes, and noncontraceptive benefits. Providers also explained to the woman that she could have it removed at any time and what she could expect before, during, and after insertion. Information about the hormonal IUS was also shared through community outreach. In Kenya, community health volunteers work with facilities to promote health behaviors and care-seeking; facilities where the hormonal IUS was available were responsible for orienting community health volunteers on the method so they could share information in communities as part of their general efforts to promote family planning. In Zambia, SM360+ oriented existing Safe Motherhood Action Groups on LARCs including the hormonal IUS, although orientations happened late in the study period. MCSP did not track data on these demand-creation activities.

## METHODS

We used a mixed-methods approach that included analysis of program monitoring data, interviews with women who received a hormonal IUS or an IUD, and qualitative focus group discussions (FGDs) with providers. IUD adopters were interviewed for comparison since adopters of hormonal IUS and IUD were both presumed to desire long-acting contraception and to be undeterred by having a device in the uterus, so we could compare other factors that influence women's decision making and experiences.

Ethical approval was received from the ethics review committees at the Johns Hopkins University School of Public Health (USA), Maseno University (Kenya), and ERES Converge (Zambia).

### Quantitative Data

In Kenya, women were enrolled in the study immediately after hormonal IUS or IUD insertion beginning in April 2017 (approximately 5 months after the initial training for mentors). All women receiving a hormonal IUS or an IUD at participating facilities were eligible for the study. Providers received training on research ethics and were given informed consent scripts to read to women after insertion. After providers obtained oral consent, they completed a short paper-based questionnaire collecting sociodemographic characteristics and contact information for follow-up. This questionnaire also collected reasons for choosing the method, what method they would have chosen if the hormonal IUS or IUD was not available, and when and how they heard about the hormonal IUS, which are questions implementers in the global hormonal IUS consultative group all agreed to collect.^21^ Consent was obtained immediately following insertion in Kenya due to initial plans to conduct follow-up interviews via short message service survey; however, the study team later opted for phone interviews instead. All women who gave a phone number were contacted for follow-up interviews. At the start of the call, women were reminded they could drop out at any point or decline to answer questions. Multiple attempts were made to call women who did not answer their phone. Phone interviews were conducted by 2 trained LARC mentors hired as temporary consultants by the study; a mentor in Migori called women in Kisumu, and a mentor in Kisumu called women in Migori to avoid the possibility of a provider interviewing her own client. RedCap v9.6.0 was used to enter data collected on the day of insertion and via phone; a single database was used to store data collected at both time points.

In Zambia, providers completed a paper-based questionnaire immediately after insertion as part of program monitoring and study recruitment. Data were collected on all women who received a hormonal IUS or IUD in participating facilities and were willing to answer the questions. Data collection started during the initial training for mentors. The questionnaire collected information similar to the one used in Kenya, but contact information was documented only for women that expressed interest in learning about the research study. The study consent process and interview were done sequentially via phone by MCSP staff. In Zambia, phone interviews were limited to women who received the hormonal IUS or IUD within 1 year after giving birth or after receiving postabortion care. The population was restricted due to the programmatic focus on maternity providers and because Society for Family Health was simultaneously introducing the hormonal IUS and collecting similar data in other public facilities in Zambia without a postpartum focus.^28^ Multiple attempts were made to call women who received a hormonal IUS or IUD postpartum or postabortion and did not answer the phone. Separate and unlinked databases were kept in Zambia for program data collected immediately after insertion and research data collected via phone. Firebase web application and Google forms were used for program and research data, respectively.

We present results based on data collected by providers at 42 facilities in Kenya from April 2017 to March 2019 and 41 facilities in Zambia from February 2017 to September 2019, although program expansion continued into additional sites after these dates. Follow-up phone interviews were conducted over 2 time periods—September 2017 and March 2019 in Kenya, and March–December 2018 and April–July 2019 in Zambia. These periods were selected based on availability of consultants and staff conducting interviews. To meet reporting requirements from the ICA Foundation, MCSP also collected the number of hormonal IUS commodities distributed to facilities and contacted facilities semiannually to obtain the total number of hormonal IUS insertions according to facility records. Data were extracted from these reports for the purpose of assessing overall uptake of hormonal IUS in program-supported facilities.

### Qualitative Data

FGDs were held with providers, with separate groups for mentors and mentees to allow different questions about the training and mentorship approach. Participants were selected by MOH staff. Each participant worked in a different facility to increase the representativeness of the sample. FGDs were conducted in English, recorded, and transcribed. In all, 2 FGDs were done with mentors (23 participants) and 4 with mentees (36 participants) in Kenya and 4 with mentors (23 participants) and 3 with mentees (15 participants) in Zambia. FGDs were held in February 2019 in Kenya and August 2018 in Zambia. Providers did not receive compensation for participation.

### Analysis

Quantitative analysis was done using Stata v14. Using data collected on the day of insertion, we ran cross-tabulations to compare hormonal IUS and IUD adopters in terms of sociodemographic characteristics, timing of insertion relative to last birth, and the method nonpostpartum adopters were switching from. We used the Pearson chi-squared test with Rao-Scott correction to adjust for clustering by facility. We also explored reasons hormonal IUS adopters chose the method; what method they would have chosen if hormonal IUS were unavailable; and where they first heard about the hormonal IUS. Using follow-up phone interviews, we cross-tabulated side effects or physical changes that hormonal IUS and IUD adopters reported providers mentioned to them on the day of insertion. In Kenya, we also examined whether the time lapse from insertion to interview seemed to affect the side effects women mentioned by restricting the analysis to women who received the hormonal IUS or IUD within 6 months before the phone call. We could not do the same in Zambia due to the small number of women participating in phone interviews. As markers of satisfaction, we looked at whether hormonal IUS adopters would recommend or have recommended the method (the question was asked differently in Kenya and Zambia) and what benefits they would mention to other women. Finally, we examined continuation rates for hormonal IUS adopters. Since there was a wide range in the time lapse from insertion to phone interviews, we restricted analysis to women interviewed 3–6 months (92–183 days) after insertion in Kenya to make the sample more homogenous. Due to the small sample size in Zambia, we were unable to restrict the sample.

FGDs were explored for information to enhance the quantitative findings, namely provider perspectives on the reasons women chose the hormonal IUS, information women received on the method, challenges to providing counseling, and client satisfaction with the method. Transcripts were coded using these themes; codes were then analyzed using principles from the One Sheet of Paper method.^29^

## RESULTS

MCSP Kenya and Zambia received 2,930 and 1,205 hormonal IUSs from ICA Foundation, respectively, with 1,985 and 428 insertions reported by facilities in Kenya and Zambia, respectively, by mid-2019. In Kenya, 289 adopters of the hormonal IUS were interviewed on the day of insertion (14.6% of hormonal IUS insertions in program-supported facilities), of which 182 (63%) participated in phone interviews. Additionally, 143 copper IUD adopters were interviewed on the day of insertion, with 87 (61%) participating in follow-up phone interviews. In Zambia, 395 adopters of the hormonal IUS were interviewed on the day of insertion (92.3% of hormonal IUS insertions); 246 were postpartum or postabortion clients, of which 40 (16%) participated in phone interviews. Additionally, 359 IUD adopters were interviewed on the day of insertion; 183 were postpartum or postabortion clients, of which 42 (23%) participated in follow-up phone interviews. Day-of-insertion interviews ranged from 1 to 47 per facility in Kenya (0–37 IUS adopters, 0–24 IUD adopters) and from 1 to 121 in Zambia (0–45 IUS adopters, 0–76 IUD adopters). The average time lapse between insertion to follow-up interview was 5 months (range 43–668 days) in Kenya and 10.4 months (range 52–773 days) in Zambia.

Totals of 1,985 and 428 insertions of the hormonal IUS were reported by facilities in Kenya and Zambia, respectively, by mid-2019.

### Characteristics of Adopters

In Kenya, no statistically significant differences were observed in the characteristics examined between hormonal IUS and IUD adopters (Table 1), although a larger proportion of hormonal IUS adopters were under age 25 (41.2% vs. 30.8%). In Zambia, statistically significant differences were apparent—more hormonal IUS adopters were under age 25 (20.8% vs. 10.3%), were never married (12.7% vs. 6.7%), and had a primary education level or less (52.7% vs. 34.0%). The proportion of high-parity (≥5 children) hormonal IUS adopters was larger in Zambia than in Kenya, but not different by method within each country. In Zambia, more adopters had given birth within 48 hours before insertion than in Kenya, although the proportion of adopters within 1 year postpartum was similar between the countries. Within Zambia, more hormonal IUS adopters received immediate postpartum insertion compared with IUD adopters (27.9% vs. 17.6%), but the difference was not statistically significant.

**TABLE 1.** Sociodemographic Characteristics and Timing of Insertion for Adopters of a Hormonal Intrauterine System or Copper-containing Intrauterine Device in Kenya and Zambia

	Kenya			Zambia		
	Hormonal IUS Adopters (N=289)	Copper IUD Adopters (N=143)	*P* Value Comparing IUS vs. IUD	Hormonal IUS Adopters (N=395)	Copper IUD Adopters (N=359)	*P* Value Comparing IUS vs. IUD
Age, years	No. (%)	No. (%)		No. (%)	No. (%)	
<20	25 (8.7)	16 (11.2)	.189	30 (7.6)	11 (3.1)	.029
20–24	94 (32.5)	28 (19.6)		52 (13.2)	26 (7.2)	
25–29	63 (21.8)	33 (23.1)		64 (16.2)	58 (16.2)	
30–34	49 (17.0)	32 (22.4)		88 (22.3)	86 (24.0)	
≥35	48 (16.6)	29 (20.3)		133 (33.7)	137 (38.2)	
Missing	10 (3.5)	5 (3.5)		28 (7.1)	41 (11.4)	
Marital status						
Married	248 (85.8)	122 (85.3)	.569	326 (82.5)	317 (88.3)	.029
Never married	32 (11.1)	19 (13.3)		50 (12.7)	24 (6.7)	
Widowed/divorced	7 (2.4)	2 (1.4)		12 (3.0)	7 (2.0)	
Missing	2 (0.7)	0 (0.0)		7 (1.8)	11 (3.1)	
Education						
None/primary	120 (41.5)	64 (44.8)	.710	212 (53.7)	122 (34.0)	.001
Secondary	98 (33.9)	41 (28.7)		127 (32.2)	137 (38.2)	
Postsecondary	66 (22.8)	34 (23.8)		49 (12.4)	69 (19.2)	
Missing	5 (1.7)	4 (2.8)		7 (1.8)	31 (8.6)	
Parity						
0	12 (4.2)	8 (5.6)	.254	9 (2.3)	11 (3.1)	.333
1–2	141 (48.8)	61 (42.7)		110 (27.9)	89 (24.8)	
3–4	87 (30.1)	42 (29.4)		103 (26.1)	118 (32.9)	
≥5	44 (15.2)	31 (21.7)		159 (40.3)	129 (35.9)	
Missing	5 (1.7)	1 (0.7)		14 (3.5)	12 (3.3)	
Timing of insertion						
Postpartum (<48 hours)	25 (8.7)	13 (9.1)	.725	110 (27.9)	63 (17.6)	.362
Postpartum (48 hours to 1 year)	152 (52.6)	71 (49.7)		119 (30.1)	102 (28.4)	
Postabortion	1 (0.4)	2 (1.4)		17 (4.3)	18 (5.0)	
Not postpregnancy	108 (37.4)	54 (37.8)		143 (36.2)	157 (43.7)	
Missing	3 (1.0)	3 (2.1)		6 (1.5)	19 (5.3)	

Abbreviations: IUD, intrauterine device; IUS, intrauterine system.

Among nulliparous and interval adopters (women whose last birth was more than 1 year prior), around half in Kenya and two-thirds in Zambia were switching from a short-acting method. In both countries, around 10% of hormonal IUS and IUD adopters were not switching from another method (Table 2).

**TABLE 2.** Interval/Nulliparous Adopters of a Hormonal Intrauterine System or Copper-containing Intrauterine Device in Kenya and Zambia Switching From Other Contraceptive Methods

		Kenya		Zambia	
		Hormonal IUS Adopters (N=108)	Copper IUD Adopters (N=54)	Hormonal IUS Adopters (N=143)	Copper IUD Adopters (N=157)
		No. (%)	No (%)	No. (%)	No. (%)
Long-acting methods	Implant	38 (35.2)	20 (37.0)	24 (16.8)	29 (18.5)
	Other IUD	4 (3.7)	0 (0.0)	7 (4.9)	11 (7.01)
Short-acting methods	Injectable	33 (30.6)	22 (40.7)	74 (51.8)	70 (44.6)
	Pills	10 (9.3)	0 (0.0)	16 (11.2)	24 (15.3)
	Condoms only	3 (2.8)	1 (1.9)	1 (0.7)	4 (2.6)
	Emergency contraceptive pills	1 (0.9)	1 (1.9)	0 (0.0)	2 (1.3)
	CycleBeads	1 (0.9)	0 (0.0)	0 (0.0)	0 (0.0)
	Traditional	1 (0.9)	0 (0.0)	3 (2.1)	0 (0.0)
	LAM	1 (0.9)	1 (1.9)	1 (0.7)	1 (0.6)
Other categories	None	10 (9.3)	4 (7.4)	15 (10.5)	15 (9.6)
	Other	0 (0.0)	0 (0.0)	2 (1.4)	1 (0.6)
	Missing	6 (5.6)	1 (1.9)	0 (0.0)	0 (0.0)

Abbreviations: IUD, intrauterine device; IUS, intrauterine system; LAM, lactational amenorrhea method.

### Reasons for Choosing a Hormonal IUS

**FIGURE 1:**
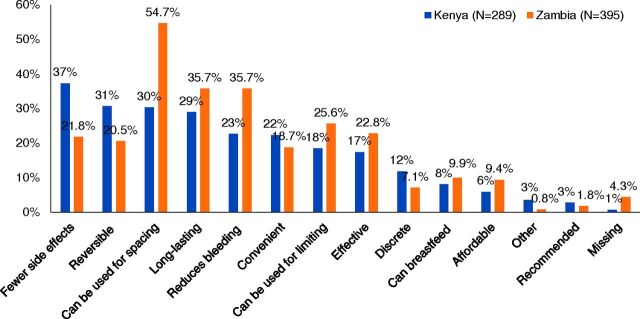
Reasons for Choosing the Hormonal Intrauterine System

Women were allowed to give multiple reasons for choosing the hormonal IUS; answers were unprompted. Women had diverse reasons for adopting the hormonal IUS. The most commonly mentioned reasons in Kenya were each mentioned by less than 40% of adopters and included the desire for fewer side effects (37%) and the facts that it is reversible (31%), can be used for spacing (30%), and is long-lasting (29%). In Zambia, the ability to use the hormonal IUS for spacing was the only reason mentioned by over half of women (55%); other top reasons included it is long-lasting (36%) and reduces bleeding (36%) (Figure 1). The ability to use it while breastfeeding was not a popular reason, but it was unsurprisingly more often mentioned by postpartum than nonpostpartum adopters (12% vs. 1% in Kenya, 15% vs. 2% in Zambia). Additional statistically significant differences for postpartum versus nonpostpartum adopters were that postpartum adopters in Kenya were more likely to mention reversibility (36% vs. 22%) and less likely to mention reduced bleeding (16% vs. 34%) and postpartum adopters in Zambia were more likely to mention spacing (60% vs. 44%).

In Kenya, 37% of women chose the hormonal IUS because they desired fewer side effects. In Zambia, 55% of women chose the method because of the ability to space pregnancies.

**FIGURE 2:**
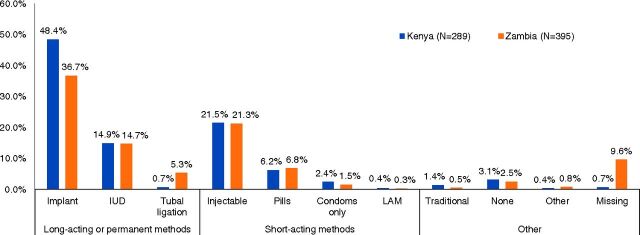
Method That Hormonal Intrauterine System Adopters Would Have Chosen if Hormonal Intrauterine System Had Not Been Available Abbreviations: IUD, intrauterine device; LAM, lactational amenorrhea method.

If the hormonal IUS had not been available in Kenya, 48% of hormonal IUS adopters would have opted for an implant, 15% an IUD, 30% a short-acting method, and 4.5% a traditional method or no contraception. In Zambia, 37% would have chosen an implant, 15% an IUD, 30% a short-acting method, and 3% a traditional method or no contraception (Figure 2).

When asked why clients chose the hormonal IUS, providers mentioned many of the same reasons as the interviewed women. The desire for fewer side effects came out strongly in the focus groups. Providers explained that women's desire for fewer side effects were, at times, tied to negative experiences using other hormonal methods, and some hormonal IUS adopters had come to the facility with the intent of switching to a method with fewer side effects. Although there was not extensive conversation about the specific side effects women sought to avoid, providers mentioned weight changes, reduced sexual desire, and cardiac effects (hypertension or palpitations). Providers reported that clients found it appealing that the hormonal IUS releases less hormone than other hormonal methods and that the hormone is localized to the uterus. These features eased women's concerns about side effects and using a hormonal method. Providers also reported women desired less bleeding to avoid discomfort, inconvenience, and disruption to their lives. The appeal of bleeding reductions came up more frequently in the Kenya interviews. There was no mention of negative reactions to reduced bleeding in any focus group.

*Maybe the hormonal level in hormonal [IUS] where the hormonal is less and it is concentrated in the uterus most of the time. Personally, as a user of hormonal I have gone into amenorrhea and that is an advantage. The hormone level is okay with me, I don't feel any palpitations, I don't have any other problem. That is the reason why I choose hormonal. So if you counsel, because with many women they tend to fear hormones, so even when you counsel and tell them that it is a 5-year and it is hormonal, unless you elaborate further that it is a hormone that concentrates mostly in the uterus and very little can be released into the system which will not affect [them], they will go for it.* —Kenya, Migori County mentor

Providers mentioned that some women like that the hormonal IUS is long-lasting but not as long-lasting as the IUD (commonly called a “copper T” in reference to the IUD's shape). This preference implies women are unaware that IUDs can be removed by choice before their full effective period. This lack of awareness may be due to inadequate counseling, misinterpreted marketing, or even difficulty experienced in accessing removals (providers in an FGD in Zambia said women reported resistance from providers when attempting to have an IUD removed before full duration). Whatever the reason, providers shared it can be challenging to overcome the perception that the IUD is only appropriate for women who want 10 years of protection, and some women still feel more comfortable with a method intended for removal within 5 years (the maximum effectiveness for the hormonal IUS at the time these programs were implemented). The 5-year duration may be especially appealing to adolescents because it aligns well with their reproductive intentions—that is, to finish school and start childbearing soon thereafter.

*The other reason is because it takes a shorter period, some women would want to have an [intrauterine device] that does not go up to 12 years like the copper T, so during the counseling when you discuss with them then they tell you, I would rather have this one because previously with the one that takes long, the copper IUD, I realized that because of ineffective counseling sometimes women are not told that whenever you want to have it removed they can just come back, that part sometimes misses that you find clients coming back to you with IUDs that were inserted 20 years ago, so for them if you talk about this, most of them say I would rather have the one with a shorter period.* —Kenya, Kisumu County, mentor

Providers also mention that the hormonal IUS can appeal to women who desire fewer visits to a facility, for convenience, cost saving, or, as 1 provider mentioned, to hide the use of contraception from her partner. Few women mentioned the ability to use the method discretely as a reason for choosing it and this reason was likewise only mentioned by a few providers. Still, it could be an important reason for a minority of women.

*I have a certain group of clients who don't want their spouses to know that they are using family planning methods yet they want the method. I have a mother who [had 10 previous births]. She just delivered yesterday and the husband is very cruel that he doesn't allow the mother to take any method. She has twelve children because she had a twin delivery previously. And all these children are alive and the spacing between them is not that good. So we counseled this mother at [antenatal care] so when she was coming for delivery, she just opted up for that method so that the husband will not know but will not take a long time as copper T.* —Kenya, Migori County, mentee

Providers mentioned additional attractive qualities that were not recorded in interviews with women, although discussion was not extensive and these may not be driving reasons for many women. These factors included a bias some women have in favor of a new method, believing it would be superior to methods that have been around a long time; the hormonal IUS does not require an incision; and removal is less painful compared with subcutaneous implants.

### Sources of Information and Content of Counseling

Most hormonal IUS adopters in Kenya heard of the method for the first time on the day it was inserted (70%), and 22.5% had heard about it from a provider during a previous visit to the facility. In Zambia, 47% heard about the method for the first time on the day of insertion, and 36% had heard about it from a provider previously (Table 3).

**TABLE 3.** Sources of Information on Hormonal Intrauterine System Among Adopters in Kenya and Zambia

	Kenya (N=289) No. (%)	Zambia (N=395)^a^ No. (%)
First heard of IUS today	201 (69.6)	187 (47.3)
Health worker another day	65 (22.5)	143 (36.2)
Community health worker	2 (0.7)	24 (6.1)
Family/friend	16 (5.5)	35 (8.9)
Other	3 (1.0)	10 (2.5)
Missing	2 (0.7)	27 (6.8)

Abbreviation: IUS, intrauterine system.

a In Zambia, more than 1 answer option was allowed.

Among adopters of the hormonal IUS, 70% in Kenya and 47% in Zambia heard of the method for the first time on the day it was inserted.

During follow-up interviews, women were asked, without prompting, what physical changes or side effects the provider mentioned at the time of insertion, and all answers were recorded. In Kenya, most women recalled the provider telling them about potential physical changes or side effects; only 3% of hormonal IUS adopters and 10% of IUD adopters reported the provider did not mention any or could not recall if the provider had mentioned any. The majority of hormonal IUS (76%) and IUD adopters (60%) said the provider mentioned changes in menstrual bleeding and 38% of hormonal IUS adopters and 39% of IUD adopters mentioned abdominal discomfort or pain. Other side effects were mentioned by a small number of women. The majority of women were told what to do if they experienced side effects (93% of hormonal IUS and 85% of IUD adopters). When restricting the analysis to women interviewed within 6 months after insertion, results did not materially change (data not shown). In Zambia, a larger proportion of women reported the provider did not mention any side effects or could not recall if the provider mentioned side effects than in Kenya (30% of hormonal IUS and 21% of IUD adopters). The difference between IUS and IUD adopters within Zambia was not statistically significant in this small sample. Similar to the results in Kenya, the most common side effects women recalled providers discussing were changes in bleeding (43% of hormonal IUS and 31% of IUD adopters) and the majority were told what to do if they experienced side effects (75% of hormonal IUS and 83% of IUD adopters), although the levels were not as high as in Kenya (Table 4).

**TABLE 4.** Information Women Reported Receiving From Provider at the Time of Insertion of Hormonal Intrauterine System or Copper-containing Intrauterine Device in Kenya and Zambia

	Kenya		Zambia	
	Hormonal IUS Adopters (N=182) No. (%)	Copper IUD Adopters (N=87) No. (%)	Hormonal IUS Adopters (N=40) No. (%)	Copper IUD Adopters (N=42) No. (%)
Physical changes/side effects mentioned				
Changes in menstrual bleeding	139 (76.4)	52 (59.8)	17 (42.5)	13 (31.0)
Vaginal discharge or infection	8 (4.4)	10 (11.5)	1 (2.5)	2 (4.8)
Headache/migraine	8 (4.4)	3 (3.4)	5 (12.5)	9 (21.4)
Nausea/vomiting	5 (2.7)	1 (1.1)	0 (0.0)	2 (4.8)
Abdominal discomfort/pain	69 (37.9)	34 (39.1)	11 (27.5)	18 (42.9)
Breast tenderness/pain	3 (1.6)	0 (0.0)	0 (0.0)	1 (2.4)
Pelvic discomfort/pain	9 (4.9)	2 (2.3)	1 (2.5)	3 (7.1)
Pain during sex	0 (0.0)	0 (0.0)	0 (0.0)	2 (4.8)
Weight gain or loss	4 (2.2)	1 (1.1)	0 (0.0)	1 (2.4)
Backache	3 (1.6)	1 (1.1)	2 (5.0)	0 (0.0)
Other	0 (0.0)	0 (0.0)	8 (20.0)	10 (23.8)
Don't know or no side effects mentioned	6 (3.3)	9 (10.3)	12 (30.0)	9 (21.4)
Missing	22 (12.1)	15 (17.2)	1 (2.5)	0 (0.0)
Told what to do if side effects occurred				
Yes	169 (92.9)	74 (85.1)	30 (75.0)	35 (83.3)
No	8 (4.4)	12 (13.8)	9 (22.5)	6 (14.3)
Missing	5 (2.7)	1 (1.1)	1 (2.5)	1 (2.4)

Abbreviations: IUD, intrauterine device; IUS, intrauterine system.

Although a large proportion of hormonal IUS adopters had not previously heard of the method, a common theme across FGDs with providers was that many women are not willing to try a method they are hearing about for the first time.

*Okay, for me, the challenge is just lack of information. The clients are not aware of the method, so when you try to tell them about the method, they will just tell you let me go and think about it, then I will come later.* —Kenya, Kisumu County, mentee

Providers shared that they found it difficult to counsel women who have no baseline awareness of the method and to overcome pervasive rumors about IUDs and contraception, including that IUDs do not stay in place and that contraception can cause cancer or infertility. The providers also shared that some women appeared interested in the method, but were unwilling to start the method that day, needing time to think and to consult their husbands. And a few providers shared stories of women who did receive the method, but then returned requesting a removal because their husbands did not like the method or did not approve of them using it. Because of these experiences, providers across most of the focus groups appealed for investments in spreading accurate information on the hormonal IUS in the community through mass media, community health workers or volunteers, or community groups so women come to facilities with awareness of the hormonal IUS, openness to using long-acting contraception, and support from their partners.

Providers appealed for investments in spreading accurate information on the hormonal IUS in the community so women come to facilities with greater awareness of it.

Although the content of counseling was infrequently discussed, in a couple of instances, providers made a direct link between counseling on side effects and women's willingness to initiate or continue using contraception. They explained that truthful conversations about side effects meant women were better prepared and were more trusting of providers if they experienced unwelcomed effects.

*Just to add-on to what she has said, like in the counseling there are times when you discover that when people are given counseling at first, the side effects never used to come out. So now we would concur with people as they are mentoring them, you come in the audience without people noticing you and listen to the on-going counseling. They will talk about the benefits as she put it but side effects are not talked about. So mothers would start shunning out family planning, but when you tell the mothers the truth of what they are going to face or to experience the first 3 months of the hormonal IUS and other IUCDs they will understand that this is what I was told and afterwards the side effects go. At least this time it's coming up bit by bit.* —Zambia, Central Province, mentor

These FGDs cannot be used to assess the quality of counseling, but it should be noted that providers often discussed feeling overworked and complained that facilities were understaffed. A few times during the discussions, providers mentioned that this situation contributed to inadequate counseling.

*Not adequately because of lack of personnel so a client will come but I'll feel I am not giving them enough time for counseling.*—Kenya, Kisumu County, mentor

### Satisfaction and Continuation Rates

In Kenya, 79% of hormonal IUS users had recommended the method to other women and 95% in Zambia would recommend the method. In terms of willingness to recommend their method, no difference was found between IUS and IUD adopters. The top benefits that IUS users in Kenya would mention to other women were reduced bleeding (49%), fewer side effects (33%), and the fact that it is long-lasting (29%). The top benefits IUS users in Zambia would mention to other women were convenience (63%), fewer side effects (34%), and high effectiveness (29%) (Table 5).

**TABLE 5.** Hormonal Intrauterine System Users That Have Recommended or Would Recommend the Method to Other Women

	Kenya, No. (%)	Zambia, No. (%)
Recommend Hormonal IUS^a^	N=182	N=40
Yes	144 (79.1)	38 (95.0)
No	34 (18.7)	1 (2.5)
Don't know	1 (0.5)	1 (2.5)
Missing	3 (1.6)	0 (0.0)
Benefits to mention^b^	N=103	N=38
Reduces bleeding	50 (48.5)	7 (18.4)
Reversible	6 (5.8)	4 (10.5)
Convenient	16 (15.5)	24 (63.2)
Fewer side effects	34 (33.0)	13 (34.2)
Discreet	4 (3.9)	1 (2.6)
Can breastfeed	5 (4.9)	1 (2.6)
Affordable	14 (13.6)	1 (2.6)
Long-lasting	30 (29.1)	8 (21.1)
Highly effective	110 (10.7)	11 (28.9)
Provider recommended	1 (1.0)	0 (0.0)
None	8 (7.8)	0 (0.0)
Other	1 (1.0)	3 (7.9)
Don't know/missing	30 (29.1)	9 (23.7)

Abbreviation: IUS, intrauterine system.

a In Kenya, women were asked *have* you recommended the method. In Zambia, women were asked *would* you recommend the method.

b In Kenya, the question was added midstudy, so the denominator includes only the women who were asked this question. The women were asked what benefits they would mention, regardless of whether they had recommended the method (yes, no, don't know, or missing to above question). In Zambia, the question was limited to women who would recommend the method (yes to above question).

In Kenya, 79% of hormonal IUS users had recommended the method to other women and 95% in Zambia would recommend the method.

Among hormonal IUS adopters in Kenya interviewed 3–6 months after insertion, 128 women (86%) were still using the method, 7 (5%) experienced an expulsion, and 11 (7%) had the method removed, and 2 responded “I don't know” to the question “Is the same IUD still in place, as far as you know?” (plus 1 with missing data). In Zambia, where the average time lapse from insertion to interview was over 10 months with a wide range for a small sample size, 31 women (79%) reported still using the method, 4 (10%) experienced an expulsion, and 4 (10%) had the method removed (plus 1 with missing data). In both countries, continuation rates were slightly higher among IUD users (94% in Kenya, 90% in Zambia) and there were fewer expulsions among IUD users, but the difference between IUS and IUD users was not statistically significant.

Continuation was very high among women who did not report experiencing a “major” problem with the hormonal IUS (93% in Kenya, 95% in Zambia) and was lower among women who did (60% in Kenya and 56% in Zambia). Among the 11 women who had the hormonal IUS removed in Kenya, 4 did not report having a major problem, and the remaining 7 women reported the following problems: cramping or pain (4), too much bleeding (2), husband disapproval (2), strings were too long (2), and irregular bleeding (1). Among 4 removals in Zambia, women reported the following problems: too much bleeding (4), dizziness (2), and cramping or pain (1). For comparison, 15 women still using the IUS in Kenya (12%) reported cramping or pain (8), too much bleeding (2), discharge (2), reduced bleeding (1), irregular bleeding (1), or husband disapproval (1). Ten women still using the IUS in Zambia (32%) reported cramping or pain (6), too much bleeding (3), too little bleeding (2), fever (2), or irregular bleeding (1).

Continuation was very high among women who did not report experiencing a “major” problem with the hormonal IUS.

Women were asked about amenorrhea separately. In Kenya, 40 women still using the IUS (31%) reported experiencing amenorrhea, with 7 saying it was a negative change. None who had the IUS removed experienced amenorrhea. In Zambia, 5 women still using the IUS (16%) reported amenorrhea, with 3 saying it was a negative change, and 1 of the 4 who had the IUS removed experienced amenorrhea saying that was a positive change (she reported dizziness as a problem).

In the focus groups, providers frequently expressed confidence that most hormonal IUS adopters were satisfied with the method because they did not return with complaints. Occasionally, providers contrasted the absence of complaints among hormonal IUS adopters with grievances heard from users of other methods.

*I feel they are satisfied because so far no one has approached me with any complaint. I have removed copper T, I have removed Implanon and Jadelle, but not hormonal [IUS]. Even those that I meet, when I ask them about the method they tell you they are comfortable.* —Zambia, Southern Province, mentor

Providers also reported hearing positive feedback from hormonal IUS users, who said they did not have any problems or were pleased to have fewer side effects than previously experienced with other methods.

*We have had interactions with them through other services that they come to seek. Maybe they have come for under-5, maybe they are sick with other ailments then you tend to interact with them and say how are you finding the hormonal [IUS] that you had received from here? They say it's just okay, I thought it would give me problems but there is nothing. Such things, so some of the women are coming out openly to say that there is no problem. And we are just encouraging them also to help us by sensitizing the women in the community because the community believes more their fellow community members than us the health practitioners.* —Zambia, Eastern Province, mentee

Providers talked about the powerful influence satisfied users can have within their circle of friends, family, and neighbors when they share their experiences using the method. Some providers reported that satisfied users in their communities led to increased interest among other women. And some providers said they had invited satisfied users to talk to other women with the intention of garnering more demand for the method.

Providers talked about the powerful influence satisfied users can have within their circle of friends, family, and neighbors.

Providers also told stories of women returning with complaints and how they were able to successfully address them with counseling and occasionally through treatment of side effects such as bleeding. With additional counseling to assuage concerns, providers reported that many women opted to continue using the method and found that the side effects eventually faded. However, a few providers gave examples of women who had bad experiences with the hormonal IUS and actively discouraged other women from using the method.

Providers also recounted cases of hormonal IUS removals. In many of the accounts mentioned, providers believed the male partners were driving the decision to have the method removed and the woman was not necessarily unhappy with the method. In 1 case, the woman even had the provider secretly reinsert the method. Providers also shared examples of women wanting or needing to have the method removed, each for a different reason, including undesired bleeding changes (spotting or excessive bleeding were mentioned), discharge, infection, and diagnosis of hypertension.

## DISCUSSION

This study provides information on characteristics of women who adopted the hormonal IUS in public facilities in 2 low-income countries. Generally, these characteristics are likely correlated with the profile of the typical family planning clientele of these facilities, which probably accounts for many differences in hormonal IUS adopters across countries and studies. A large proportion of Kenyan adopters in this study and a prior study of postpartum adopters at a public facility in Kenya^11^ were young (41% under 25 years in this study and 51% in the previous study) and had low educational attainment (41.5% with primary level or less in this study and 61.5% in the previous study). In this study, adopters were older in Zambia (47% were ≥35 years) and educational attainment was lower (54% with primary or less). Adopters recruited at hospitals in a prior study in Ghana were older than those in either Kenya study, with a similar age distribution to our population in Zambia (31% age ≥35 years) but were more highly educated than adopters in Kenya or Zambia.^15^ Adopters recruited from social franchise and outreach sites in a prior study in Nigeria were even older (47% age ≥35 years) and highly educated (70% had secondary or higher education).^13^ Adopters were mostly married in both countries in this study as well as in the previous studies in Kenya, Ghana, and Nigeria, suggesting that specific outreach may be necessary to increase use among unmarried women in many contexts.

Comparisons of hormonal IUS to IUD adopters at the same facilities allows additional insight into whether hormonal IUS appeals to a different set of women. The results from this study suggest that hormonal IUS may appeal more to young women than the IUD since a greater proportion of hormonal IUS adopters were under age 25 in both countries (although the differences was not statistically significant in Kenya). More adopters of the hormonal IUS in Zambia were never married and had lower educational attainment, which may be attributable to the differences in the age profile. Provider testimonies that shorter duration of effectiveness is a favorable attribute of the hormonal IUS could explain why the method may be more appealing to younger women than IUDs, as they may want to become pregnant in the near to intermediate term.

A substantial proportion of women choosing the hormonal IUS (35% in Kenya and 33% in Zambia) would have opted for a short-acting or traditional method or none at all, if the hormonal IUS had not been an option. This finding is similar to what Hubacher et al.^11^ found among postpartum adopters in Kenya (30.5% would have chosen a short-acting method instead) and higher than what Eva et al.^13^ found among social franchise and outreach clients in Nigeria (20% would have chosen a short-acting, traditional, or no method). Both previous studies concluded that some women are willing to try the hormonal IUS because of its features and do not see it as interchangeable with other long-acting methods. We reached the same conclusion, among a broader population of hormonal IUS adopters accessing contraception at public facilities, adding to the evidence that the hormonal IUS has features that appeal to women that they may not find in other LARCs. Adding hormonal IUS to the method mix could increase the proportion of women opting for a LARC. At the same time, some adopters were already using a long-acting method (39% of nonpostpartum adopters in Kenya and 22% in Zambia were switching from another long-acting method, mainly implants), suggesting that some women using LARCs are not completely satisfied with their methods and would prefer to try a LARC with different features.

Many adopters would have opted for a short-acting or traditional method or none at all, if the hormonal IUS had not been an option.

As in other studies, women gave a range of reasons for choosing the hormonal IUS. A desire for fewer side effects emerged at the top. Among adopters we interviewed, 37% in Kenya mentioned hormonal IUS having fewer side effects than other methods and 23% specifically mentioned reduced bleeding. In Zambia, 22% mentioned fewer side effects and 36% specifically mentioning reduced bleeding. Users of the hormonal IUS also reported that fewer side effects and reduced bleeding were benefits they would mention to other women when recommending the method. A desire for fewer side effects was also a popular reason for women in past studies.^11^^,^^13^^,^^15^^,^^30^ The fact that it can be used discretely was not mentioned as frequently by women in this study (12% in Kenya, 7% in Zambia) compared with past studies in Kenya (23%)^11^ or Nigeria (42%).^13^ However, it may be an important reason for a minority of women based on stories shared by providers during FGDs. Given that some women like the long, but not too long, duration of effectiveness and that reversibility was a popular reason, the immediate return to fertility after IUS removal may be an important consideration to emphasize during counseling and marketing, especially with younger clients. Overall, women gave diverse reasons for choosing the method in this study and previous ones, suggesting that it can appeal to women wishing to adopt contraception for a broad range of reasons.

Women gave diverse reasons for choosing the method, suggesting that it can appeal to women wishing to adopt contraception for a broad range of reasons.

Many hormonal IUS adopters had never previously heard of the method. Providers reported that lack of awareness of the method among their clients handicapped uptake, but they saw potential for further growth in interest in hormonal IUS if information on the method could be disseminated through community channels. Although few adopters reported hearing about the method from family or friends, providers believed that satisfied users can be powerful assets to encourage other women to try the method. The critical role of satisfied users to increasing demand for IUDs has been shown in past research.^13^^,^^31^ The qualitative interviews also reinforce the importance of educating and engaging male partners, since they often influence or even determine women's decision to adopt or continue using contraception. Yet we recognize the need for nuance in male engagement. For a subset of women, it may be important to be able to use contraception covertly and counseling couples together could have unintended consequences.^32^

Most women reported being told what to do if they experienced side effects, and many recalled being told about bleeding changes and abdominal pain, although other side effects were rarely recalled and may not have been emphasized or mentioned at all. Providing clients with information about contraceptive methods in general and their chosen method in particular is associated with improved continuation.^33^ A few providers talked about this link during FGDs, while at the same time, providers suggested counseling may not have always been as high quality as they thought was ideal.

Despite some indications that the quality of counseling before insertion was not always optimal, user satisfaction levels were high, as evidenced by the high proportion of users who would recommend the hormonal IUS to other women. We also found a high level of continuation among women in Kenya 3–6 months after insertion (86%), on par with the rate seen in a previous study in Kenya at 12 months (89%).^14^ Continuation was slightly lower in Zambia (78%), but the sample was extremely small and some women were interviewed close to 2 years after insertion. Even among women that reported experiencing what they perceived to be a major problem, the majority continued to use the method. Additionally, only a minority of women experiencing amenorrhea felt negatively about that change (in Kenya; the numbers were very small in Zambia). Earlier studies also showed that amenorrhea or reduced bleeding may be welcomed by many users, but be an unwelcome change for some.^13^^,^^34^ Good counseling before insertion is critical to ensure that a woman selects a method that aligns with her preferences, and it is also an important aspect of follow-up care to reduce discontinuation. Providers shared experiences with successfully addressing concerns among women returning to facilities citing problems with the method. However, we did not collect data from women who experienced problems on why they continued to use the method and how interactions with providers contributed to that decision.

User satisfaction levels were high, as evidenced by the high proportion of users who would recommend the hormonal IUS to other women.

Uptake of the hormonal IUS was higher in Kenya than Zambia. Two prolonged health care worker strikes (December 2016 to March 2017 and June–November 2017) disrupted services in Kenya during the study period, but demand quickly recovered after each strike ended. The difference between countries may be partially explained by general trends in contraceptive use in each country. The modern contraceptive prevalence rate (mCPR) was 34.1% in Zambia in 2018.^35^ In Kenya, mCPR was 39.1% in 2014^36^ and seems to have increased in the intervening years, with mCPR being 44.6% across 11 counties included in the Performance Monitoring and Accountability 2020 Survey conducted in 2018.^37^ IUD use is low in both countries, but higher in Kenya (2.3% in 2014^36^) than in Zambia, where it declined from 0.9% in 2014^38^ to 0.5% in 2018.^35^ The difference in uptake between countries may also be explained by implementation factors, including the focus on training maternity providers in Zambia and the wide geographic spread of implementation sites in Zambia across 4 provinces, presenting challenges to managing commodities and monitoring the mentorship process. Assessing the contribution of these factors to uptake, however, is beyond the scope of this article.

For context, we compared the number of IUS insertions in project-supported facilities to MOH data from the same facilities over the same period. In Kenya, we reported 1,985 IUS insertions over a period when approximately 8,000 total intrauterine insertions (IUS + IUDs) and 37,000 implant insertions were reported by the same facilities. In Zambia, we reported 428 IUS insertions over a period when approximately 2,700 total intrauterine insertions (IUS + IUDs) and 16,000 implant insertions were reported by the same facilities. (MOH numbers in Zambia are underreported because we were unable to get data from 7 facilities, plus 6 facilities reported fewer intrauterine insertions than the number interviewed for this study.)

We found wide variation in uptake across facilities. In some participating facilities, no IUS insertions were reported. We know from project reports and focus groups that some trained providers were transferred to different facilities and some did not yet feel comfortable in their skills. As mentioned above, providers reported challenges in offering a new method that women were not familiar with. From the MOH data, we also see a wide range across participating facilities in terms of total LARC insertions. Adoption of new practices is often uneven, although it could be improved with careful tracking and by quickly responding when providers are transferred or are found to not be adequately skilled. Investments in widespread demand creation could also support more even adoption.

### Limitations

Study limitations include that findings may not be representative of all women receiving a hormonal IUS; in Kenya, only around 15% of women receiving the IUS were interviewed at the time of insertion, either because women refused to participate or providers did not take the time to enroll all eligible women. We do not know if interviewed adopters were different from those not interviewed in ways that might affect the results presented in this article. In both countries, the study conducted follow-up phone interviews with a minority of adopters because many women were unable or unwilling to share phone numbers or did not answer their phones after multiple attempts to reach them. Data from follow-up interviews are thus likely biased toward women who have greater phone access, including women with a higher income. We did not track how many insertions were done by providers who were still in training, but it is likely to be many because there was a long training and mentorship process before certification. It is possible that clients' experience of care, satisfaction, and continuation may shift over time as the number of more experienced providers increases. In Kenya, we conducted fewer interviews with IUD adopters than IUS adopters, which affected our ability to detect statistically significant differences between the groups. Results from follow-up interviews are subject to recall bias. In addition, women were interviewed at very different time points after insertion, so data on continuation have to be interpreted cautiously. Qualitative interviews were conducted with providers only, and there may have been a social desirability bias, especially since program staff were in attendance to assist the qualitative interviews and interviews were conducted on project premises. Also, MOH may have identified providers that were high performing or held more positive views on the training and mentorship experience to participate in the interviews.

## CONCLUSION

Expanding access to the hormonal IUS through the public sector as part of a range of contraceptive choices shows promise to increase overall use of contraception and continuation in both Kenya and Zambia. Supporting providers to acquire skills in both counseling and insertion is essential and efficiencies can be gained if integrated within efforts to fill LARC competency gaps, rather than offering stand-alone hormonal IUS training, although proficient LARC providers only need tailored support to add this method to their skillset. Ensuring that training includes skills for insertion in interval, postabortion, and immediate postpartum services can improve the diversity of clients who benefit from this method and requires only a marginal additional investment. Efforts to strengthen availability of the services should not ignore demand-related efforts and engaging directly with various communities around the availability of a new LARC option with its own method characteristics. These communities could include youth groups and channels for unmarried women as well as men. With global donors and multilateral agencies planning to introduce hormonal IUS as part of offerings for commodity donations to countries, there will be more opportunities for women in LMICs to access and voluntarily opt for this method.

## Notes

**Peer Reviewed**

**First published online:** March 16, 2021.
